# Construction of a Nomogram Discriminating Malignancy-Associated Membranous Nephropathy From Idiopathic Membranous Nephropathy: A Retrospective Study

**DOI:** 10.3389/fonc.2022.914092

**Published:** 2022-07-14

**Authors:** Ting Wang, Wei Yu, Feng Wu, Yiding Zhang, Jin Shang, Zhanzheng Zhao

**Affiliations:** ^1^ Department of Nephrology, the First Affiliated Hospital of Zhengzhou University, Zhengzhou, China; ^2^ Laboratory Animal Platform of Academy of Medical Sciences, Zhengzhou University, Zhengzhou, China

**Keywords:** membranous nephropathy, malignancy-associated membranous nephropathy, malignancy, Cysc, discriminant model

## Abstract

**Background:**

Based on the etiology, membranous nephropathy (MN) can be categorized into idiopathic membranous nephropathy (IMN) and secondary membranous nephropathy. Malignancy-associated membranous nephropathy (MMN) is a common type of secondary MN. Its incidence is only second to that of lupus nephritis. As the treatment and prognosis of MMN differ significantly from those of other MNs, the identification of MMN is crucial for clinical practice. The purpose of this study was to develop a model that could efficiently discriminate MMN, to guide more precise selection of therapeutic strategies.

**Methods:**

A total of 385 with IMN and 62 patients with MMN, who were hospitalized at the First Affiliated Hospital of Zhengzhou University between January 2017 and December 2020 were included in this study. We constructed a discriminant model based on demographic information and laboratory parameters for distinguishing MMN and IMN. To avoid an increased false positivity rate resulting from the large difference in sample numbers between the two groups, we matched MMN and IMN in a 1:3 ratio according to gender. Regression analysis was subsequently performed and a discriminant model was constructed. The calibration ability and clinical utility of the model were assessed *via* calibration curve and decision curve analysis.

**Results:**

We constructed a discriminant model based on age, CD4^+^ T cell counts, levels of cystatin C, albumin, free triiodothyronine and body mass index, with a diagnostic power of 0.860 and 0.870 in the training and test groups, respectively. The model was validated to demonstrate good calibration capability and clinical utility.

**Conclusion:**

In clinical practice, patients demonstrating higher scores after screening with this model should be carefully monitored for the presence of tumors in order to improve their outcome.

## Introduction

Membranous nephropathy (MN), the most common cause of nephrotic syndrome in adults, is pathologically characterized by diffuse glomerular basement membrane thickening with subepithelial immune complex deposition. The immune complex is composed of immunoglobulin G, related antigens, complement components, and the membrane attack complex. MN can be categorized into idiopathic membranous nephropathy (IMN) and secondary membranous nephropathy according to the etiology. The most common cause of secondary membranous nephropathy is autoimmune disease, mainly systemic lupus erythematosus. Malignancy-associated membranous nephropathy (MMN) ranks second. Other causes include infections, drugs, and heavy metals (1).

In 1996, Lee et al. (2) first proposed the presence of a certain relationship between nephrotic syndrome and malignancy. In this context, the relationship between malignancy and renal disease has always concerned clinicians. Studies have found that the prevalence of malignant tumors in MN patients is 6–22% (3). The renal pathology of MMN is usually characterized by deposition of IgG1 and IgG2 without IgG4, while that of IMN mainly involves IgG4 deposition (4, 5). In recent years, the role of podocyte antigens has been increasingly recognized in MN. Studies have shown that patients with MN who presented PLA2R-negative but THSD7A or NELL1-positive are more likely to suffer from malignancies (6–8).

As the treatment of MMN differs completely from that of IMN, immunosuppressive therapy recommended for IMN may exacerbate the malignancy; accurate diagnosis of MMN is therefore crucial. There are currently three criteria for MMN: i. the clinical symptoms and pathological features of renal disease are relieved after complete tumor remission, ii. pathological examination of renal tissue demonstrates positivity for the tumor antigen or antibody, and iii. recurrence or worsening of kidney disease is observed after tumor recurrence. However, the clinical application of the abovementioned standards is relatively difficult. As many antigens remain undiscovered, obtaining an early warning is currently difficult (9). Researchers have been working on the differentiation of MMN and IMN. Most of the current research focuses on renal pathology, including the deposition of IgG1, IgG2, and IgG4, and the renal expression of new molecular markers such as PLA2R, THSD7A, and NELL1 (10, 11).

In this study, we aimed to build a simple and highly feasible differential model based on demographic characteristics and laboratory indicators, to identify MMN at an early stage and achieve the purpose of early detection, diagnosis, and treatment.

## Material and Methods

### Patient Selection and Ethical Approval

We included patients with MN who were admitted to the First Affiliated Hospital of Zhengzhou University between January 2017 and December 2020. This study was approved by the First Affiliated Hospital of Zhengzhou University Ethics Review Committee (ZY-2021-0008). The inclusion criteria for IMN were as follows: i. patients aged 18–80 years, ii. initial onset of MN, and iii. diagnosed with MN by renal biopsy. The exclusion criteria were: i. prior history of administration of corticosteroids or immunosuppressors, and ii. presence of secondary membranous nephropathy, such as MN associated with autoimmune disease, cancer, infection, or drug toxicity. A total of 71 patients having both MN and malignancy were included in the study.

**Figure 1:**
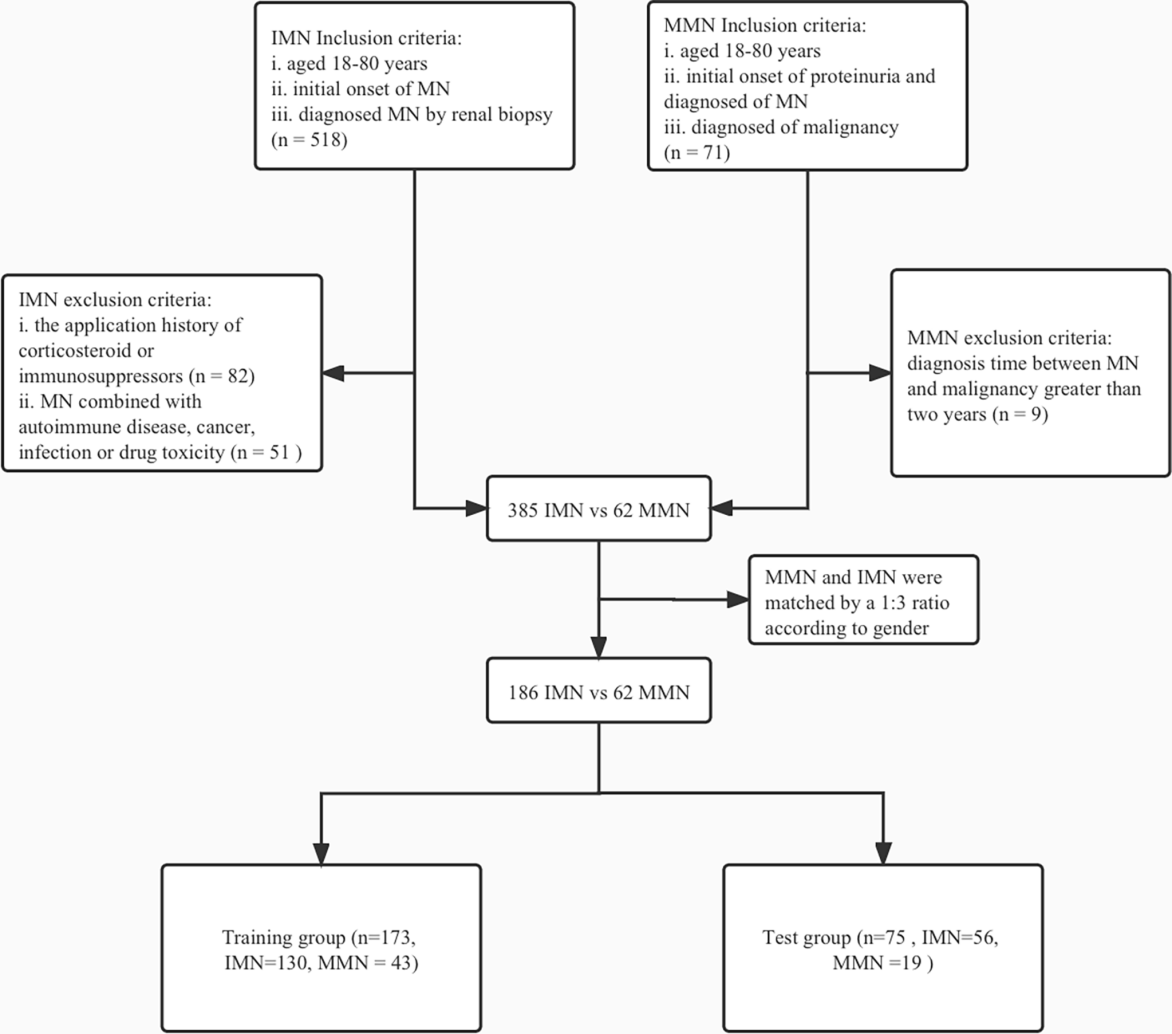
Flowchart of enrollment and exclusion in training and test group. IMN, idiopathic membranous nephropathy; MN, membranous nephropathy; MMN, malignancy-associated membranous nephropathy.

Due to the current lack of clear criteria for MMN, contemporary diagnoses of MN and malignancy are generally accepted as a surrogate for the association between these two diseases (12). Therefore, our criteria for inclusion of patients with MMN were as follows: i. aged 18–80 years, ii. initial onset of proteinuria and diagnosed as MN, and iii. diagnosed of malignancy within 2 years before or after the diagnosis of MN; patients diagnosed beyond this time range were excluded. Based on the criteria described, 385 cases of IMN and 62 cases of MMN were included in the study cohort (**Figure 1**).

### Data Collection

Demographic, clinical, and pathological baseline data were obtained at the time of renal biopsy or when patients were first admitted for nephrotic syndrome. The demographic information included age, gender, body mass index, systolic blood pressure, diastolic blood pressure, history of hypertension, and family history of malignancy. Clinical indices included white blood cell and platelet counts, and levels of hemoglobin, alanine aminotransferase, aspartate aminotransferase, serum creatinine (Scr), uric acid, cystatin C (Cysc), albumin (ALB), total cholesterol, triglycerides (TG), high density lipoprotein (HDL), low density lipoprotein (LDL), β2-microglobulin, α1-microglobulin, C-reactive protein, complement 3, complement 4, free triiodothyronine (FT3), free thyroxine, thyroid stimulating hormone, M-type phospholipase A2 receptor (PLA2R), and 24-h uric total protein (24hTP), CD4^+^ T cell (CD4) and CD8^+^ T cell (CD8) counts, the CD4/CD8 ratio, estimated glomerular filtration rate (eGFR) and erythrocyte sedimentation rate. Data regarding tubular atrophy and interstitial fibrosis were collected as pathological indicators.

### Statistical Analysis

#### Data Processing

The proportions of missing values were as follows: 24hTP had 1.1% missing values; Cysc, HDL, and LDL had 2.2% missing values; and CD4 and CD8 had 4.3% missing values. In order to address the issue of missing data, achieve maximum statistical power, and reduce bias, we used multiple imputation method to impute missing values (13). The means, medians, and percentages were used to characterize variables in each group. Categorical data have been presented as percentages. Continuous variables with or without normal distribution have been presented as the mean ± standard deviation (SD) or median (quartile 1, quartile 3). Two groups of baseline data were compared using the unpaired Student’s t- or Mann–Whitney U tests for quantitative data and the chi-square test analysis for qualitative data. Due to the large difference in sample size between the MMN and IMN groups, direct comparison and modeling could have exaggerated the difference between the two groups, increased the false positivity rate, and led to unreliable regression results. Therefore, we matched MMN and IMN groups in a 1:3 ratio using the propensity score method. Our preliminary analysis showed no statistical difference between the two groups in terms of gender. We matched groups based on gender using R software to ensure that the selected subjects were comparable in terms of clinical characteristics.

#### Model Development

We used the Akaike information criterion to construct the best fitting model by stepwise regression. A discriminant model was developed to differentiate between patients with IMN and MMN, based on statistically significant clinical characteristics. The receiver operating characteristic curve was plotted and the area under the curve was calculated to evaluate the validation efficiency of the model. The model was verified by resampling based on the original data set using the bootstrapping method (14). A calibration curve was constructed to evaluate the calibration ability of the model. After comprehensively evaluating the performance of the model, the best model was obtained; a nomogram that could be conveniently used in the clinic was then constructed. The coefficient of determination, R^2^, evaluated the degree of fit of the regression equation. Decision curve analysis was performed to determine the clinical utility of the discriminant model at different threshold probabilities by quantifying the net benefit (15). All statistical analysis was performed by R software, version 4.0.2. A P value of < 0.05 was considered statistically significant. Our manuscript for developing a multivariate prediction model is based on the TRIPOD statement (16).

## Results

### Baseline Characteristics of Malignancy-Associated Membranous Nephropathy and Idiopathic Membranous Nephropathy

The demographic, clinical, and pathological baseline characteristics of IMN (n=385) and MMN (n=62) are presented in **Table 1**. The median ages for IMN and MMN were 49.0 (37. 0, 57.0) years and 59 (53.2, 67.0) years, respectively. Compared with patients of IMN, those with MMN tended to be older and had higher Scr, Cysc, and ALB levels. They also had lower eGFR, TG, PLA2R levels, and CD4, CD8 counts. The gender ratio between the two groups was not statistically different (p=0.989). There were no significant differences between the two groups in terms of history of hypertension, family history of malignancy, and renal tubular atrophy/interstitial fibrosis. In order to increase the reliability of the regression model, we reduced the difference in the number of samples between IMN and MMN groups. We matched the IMN and MMN groups according to gender; the baseline information after matching is shown in **Table 2**.

**Table 1:** Baseline characteristics for all patients with IMN and MMN.

	IMN	MMN	p value
	N=385	N=62	
Gender:			0.989
Male	232 (60.3%)	38 (61.3%)	
Female	153 (39.7%)	24 (38.7%)	
Age[years]	49.0 [37.0;57.0]	59.0 [53.2;67.0]	<0.001
BMI[kg/m2]	25.0 [23.1;27.9]	24.2 [22.2;26.1]	0.023
SBP[mmHg]	132 [123;140]	134 [125;142]	0.227
DBP[mmHg]	83.0 [76.0;90.0]	82.5 [75.0;89.0]	0.543
History of hypertension:			0.429
NO	237 (61.6%)	42 (67.7%)	
YES	148 (38.4%)	20 (32.3%)	
Family history of malignancy:			0.157
NO	354 (91.9%)	53 (85.5%)	
YES	31 (8.05%)	9 (14.5%)	
WBC[109/L]	6.30 [5.20;7.80]	6.39 [5.50;7.91]	0.592
PLT[109/L]	234 [194;274]	226 [178;284]	0.692
Hb[g/L]	127 [117;140]	130 [113;139]	0.693
ALT[U/L]	15.0 [12.0;23.0]	16.0 [12.2;20.8]	0.872
AST[U/L]	19.0 [15.0;24.0]	20.0 [16.0;23.0]	0.438
Scr[μmol/L]	70.0 [58.0;81.0]	71.5 [62.0;92.8]	0.063
UA[μmol/L]	324 [267;387]	324 [263;372]	0.848
eGFR(ml/min/1.73m2)	101 [89.7;113]	89.2 [74.3;99.9]	<0.001
Cysc[mg/L]	0.96 [0.83;1.12]	1.18 [1.02;1.50]	<0.001
ALB[g/L]	23.9 [20.3;27.9]	26.4 [21.7;30.2]	0.037
TCHO [mmol/L]	6.83 [5.67;8.06]	6.48 [4.68;7.40]	0.055
TG [mmol/L]	2.08 [1.44;3.01]	1.73 [1.23;2.31]	0.01
HDL [mmol/L]	1.32 [1.08;1.58]	1.30 [1.08;1.59]	0.897
LDL [mmol/L]	4.68 [3.40;6.24]	4.39 [2.95;5.56]	0.112
β2-MG[mg/L]	1.58 [1.15;2.21]	2.10 [1.54;3.36]	<0.001
α1-MG[mg/L]	34.5 [28.0;42.0]	38.0 [30.2;43.8]	0.159
CRP[mg/L]	1.10 [0.00;2.27]	1.69 [0.72;3.07]	0.003
ESR[mm/h]	36.0 [18.0;57.0]	34.0 [15.0;70.5]	0.751
C3[g/L]	1.32 [1.15;1.50]	1.21 [1.09;1.45]	0.084
C4[g/L]	0.30 [0.25;0.36]	0.30 [0.26;0.35]	0.689
FT3[pmol/L]	3.89 [3.30;4.44]	4.13 [3.39;4.59]	0.224
FT4[pmol/L]	10.8 [9.66;12.1]	10.7 [9.70;11.9]	0.815
TSH[μIU/mL]	3.52 [2.24;5.30]	3.37 [2.17;5.54]	0.754
PLA2R[RU/mL]	48.8 [12.3;135]	25.4 [2.42;116]	0.048
24hTP[g]	4.64 [2.94;6.80]	5.12 [2.65;7.43]	0.496
CD4[/μl]	804 [580;1108]	679 [368;912]	0.009
CD8[/μl]	460 [324;625]	413 [289;540]	0.044
CD4/CD8	1.74 [1.22;2.40]	1.66 [1.18;2.34]	0.445
^a^Tubular atrophy:			0.202
0	115 (29.9%)	9 (17.6%)	
1	68 (17.7%)	7 (13.7%)	
2	85 (22.1%)	12 (23.5%)	
3	70 (18.2%)	13 (25.5%)	
4	47 (12.2%)	10 (19.6%)	
^b^Interstitial fibrosis:			0.116
0	103 (26.8%)	8 (15.7%)	
1	22 (5.71%)	1 (1.96%)	
2	64 (16.6%)	7 (13.7%)	
3	196 (50.9%)	35 (68.6%)	

IMN, idiopathic membranous nephropathy; MMN, malignancy-associated membranous nephropathy; BMI, body mass index; SBP, systolic blood pressure; DBP, diastolic blood pressure; WBC, white blood cell; PLT, platelet; Hb, hemoglobin; ALT, Alanine aminotransferase; AST, Aspartate aminotransferase; Scr, serum creatinine; UA, uric acid; eGFR, estimated glomerular filtration rate; Cysc, Cystatin C; ALB, albumin; TCHO, total cholesterol; TG, triglycerides; HDL, high density lipoprotein; LDL, low density lipoprotein; CRP, C-reactive protein; ESR, erythrocyte sedimentation rate; C3, complement 3; C4, complement 4; FT3, free triiodothyronine; FT4, free thyroxine; TSH, thyroid stimulating hormone; PLA2R, M-type phospholipase A2 receptor; 24hTP, 24h uric total protein; CD4, CD4^+^ T cells count; CD8, CD8^+^ T cells count. ^a^Tubular atrophy: 0 Vacuole and granular degeneration of renal tubular epithelial cells; 1 Vacuole, granular degeneration of renal tubular epithelial cells, and shedding of brush borders; 2 Individual tubular atrophy <1%; 3 Focal atrophy <10%, 4 Focal atrophy >10%. ^b^Interstitial fibrosis: 0 No obvious lesions in the renal interstitium; 1 Renal interstitial lymphatic and mononuclear cell infiltration; 2 Renal interstitial lymphatic and mononuclear cell infiltration with edema; 3 Renal interstitial lymphatic and mononuclear cell infiltration with fibrosis.

**Table 2:** Baseline characteristics of IMN and MMN patients included in the model.

	IMN	MMN	p v*al*ue
	N=186	N=62	
Gender:			1
Male	114 (61.3%)	38 (61.3%)	
Female	72 (38.7%)	24 (38.7%)	
Age[years]	50.0 [37.2;57.0]	59.0 [53.2;67.0]	<0.001
BMI[kg/m2]	25.1 [23.1;27.6]	24.2 [22.2;26.1]	0.027
SBP[mmHg]	132 [123;142]	134 [125;142]	0.397
DBP[mmHg]	84.0 [76.0;90.0]	82.5 [75.0;89.0]	0.263
History of hypertension:			0.938
NO	123 (66.1%)	42 (67.7%)	
YES	63 (33.9%)	20 (32.3%)	
Family history of malignancy:			0.41
NO	168 (90.3%)	53 (85.5%)	
YES	18 (9.68%)	9 (14.5%)	
WBC[109/L]	5.90 [5.00;7.08]	6.39 [5.50;7.91]	0.061
PLT[109/L]	228 [196;273]	226 [178;284]	0.768
Hb[g/L]	126 [117;138]	130 [113;139]	0.906
ALT[U/L]	15.0 [12.0;21.8]	16.0 [12.2;20.8]	0.762
AST[U/L]	18.0 [15.0;23.0]	20.0 [16.0;23.0]	0.133
Scr[μmol/L]	70.0 [58.0;80.0]	71.5 [62.0;92.8]	0.078
UA[μmol/L]	324 [264;385]	324 [263;372]	0.982
eGFR(ml/min/1.73m2)	102 [91.0;112]	89.2 [74.3;99.9]	<0.001
Cysc[mg/L]	0.93 [0.82;1.08]	1.18 [0.96;1.48]	<0.001
ALB[g/L]	23.4 [20.2;27.9]	26.4 [21.7;30.2]	0.02
TCHO [mmol/L]	6.69 [5.76;7.90]	6.48 [4.68;7.40]	0.098
TG [mmol/L]	1.92 [1.32;2.94]	1.73 [1.23;2.31]	0.14
HDL [mmol/L]	1.32 [1.12;1.53]	1.32 [1.10;1.62]	0.791
LDL [mmol/L]	4.65 [3.48;6.10]	4.36 [3.03;5.61]	0.115
β2-MG[mg/L]	1.54 [1.13;2.11]	2.04 [1.51;3.36]	<0.001
α1-MG[mg/L]	35.0 [28.0;43.0]	38.0 [30.2;45.5]	0.263
CRP[mg/L]	1.15 [0.40;2.27]	1.63 [0.70;2.99]	0.023
ESR[mm/h]	33.0 [18.0;57.0]	35.0 [15.2;71.5]	0.478
C3[g/L]	1.25 [1.10;1.45]	1.19 [1.07;1.45]	0.334
C4[g/L]	0.30 [0.26;0.35]	0.30 [0.26;0.36]	0.749
FT3[pmol/L]	3.87 [3.27;4.35]	4.25 [3.47;4.71]	0.028
FT4[pmol/L]	10.8 [9.71;11.8]	10.6 [9.57;11.6]	0.397
TSH[μIU/mL]	3.64 [2.55;5.63]	3.66 [2.22;5.88]	0.536
PLA2R[RU/mL]	53.7 [13.0;116]	25.4 [2.42;116]	0.077
24hTP[g]	4.68 [3.16;6.95]	5.09 [2.64;7.21]	0.844
CD4[/μl]	924 [671;1167]	682 [369;942]	<0.001
CD8[/μl]	458 [333;607]	404 [288;552]	0.017
CD4/CD8	2.02 [1.45;2.60]	1.62 [1.17;2.30]	0.002
Tubular atrophy:			0.001
0	60 (32.3%)	10 (16.1%)	
1	29 (15.6%)	8 (12.9%)	
2	55 (29.6%)	14 (22.6%)	
3	35 (18.8%)	19 (30.6%)	
4	7 (3.76%)	11 (17.7%)	
Interstitial fibrosis:			0.116
0	53 (28.5%)	9 (14.5%)	
1	8 (4.30%)	2 (3.23%)	
2	30 (16.1%)	10 (16.1%)	
3	95 (51.1%)	41 (66.1%)	

### Incidence and Cancer Types in Patients With Membranous Nephropathy

The cancer prevalence in patients with MN was 16.1%; this increased significantly with age (**Table 3**). The types of cancer associated with MN are shown in **Table 4**. The most common localization and pathological types of MMN were lung adenocarcinoma (14 cases, 22.6%) and papillary thyroid carcinoma (12 cases, 19.4%). MN-related gastrointestinal tumors included rectal (4 cases), colonic (3 cases), gastric (3 cases), and esophageal cancers (1 case). In addition to solid tumors, there were non-solid tumors such as multiple myeloma (2 cases) and lymphoma (1 case).

**Table 3:** Age distribution of cancer cases in MN patients.

Age	Male			Female			Total		
	IMN	MMN	%	IMN	MMN	%	IMN	MMN	%
18-54	153	9	5.8	103	9	8.7	256	18	7.0
55-64	51	14	27.4	34	9	26.5	85	23	27.1
>=65	28	15	53.5	16	6	37.5	44	21	47.8
Total	232	38	16.4	153	24	15.7	385	62	16.1

**Table 4:** Types of cancer among 62 patients with MMN.

Localization of tumor	Histology	N
Lung	Adenocarcinoma	14
	Small cell carcinoma	1
	Squamous cell carcinoma	1
Thyroid	Papillary carcinoma	12
Rectum	Adenocarcinoma	4
Prostate	Adenocarcinoma	4
Stomach	Adenocarcinoma	3
Colon	Adenocarcinoma	3
Cervix	Squamous cell carcinoma	3
Bladder	Adenocarcinoma	3
Thymus	Thymoma	3
Multiple myeloma		2
Kidney	Adenocarcinoma	2
Lymphoma		1
Throat	Squamous cell carcinoma	1
Breast	Ductal carcinoma	1
Esophagus	Squamous cell carcinoma	1
Endometrial lining	Adenocarcinoma	1
Liver	Hepatocellular carcinoma	1
Pancreas	Papillary carcinoma	1

### Six Potential Indicators Were Used to Build the Predictive Model

Owing to limited data regarding patients with MMN, we performed internal validation with the dataset instead of external validation. Participants were randomly divided into training (n=173) and test (n=75) groups. As shown in **Table 5**, the two groups did not differ significantly in terms of baseline data. The odds ratios and p value of logistic regression are shown in **Table 6**. Using univariate regression analysis, 15 statistically significant (p < 0.05) potential predictors were screened out from 36 variables in the training group. Age, Cysc, ALB, FT3, and CD4 met the criteria after excluding co-linearity among variables using multivariate regression analysis. The multivariate logistic regression results showed that the p value of BMI was 0.062, and the odds ratios (95% CI) value was (0.817, 1.005). Considering that BMI has a p-value close to 0.05 and has important clinical value in multiple previous disease states, BMI was included in the model. The final equation was as follows:

**Table 5:** No difference of the baseline characteristics between the training group and test group.

	Training group	Test group	p value
	N=173	N=75	
Gender:			0.484
Male	109 (63.0%)	43 (57.3%)	
Female	64 (37.0%)	32 (42.7%)	
Age[years]	53.0 [45.0;61.0]	50.0 [38.0;59.5]	0.145
BMI[kg/m2]	25.2 [23.3;27.5]	24.2 [22.0;27.0]	0.045
SBP[mmHg]	133 [125;142]	132 [124;140]	0.577
DBP[mmHg]	83.0 [76.0;90.0]	86.0 [76.5;90.0]	0.282
History of hypertension:			0.682
NO	117 (67.6%)	48 (64.0%)	
YES	56 (32.4%)	27 (36.0%)	
Family history of malignancy:			1
NO	154 (89.0%)	67 (89.3%)	
YES	19 (11.0%)	8 (10.7%)	
WBC[109/L]	6.00 [5.10;7.50]	6.00 [5.33;6.90]	0.917
PLT[109/L]	227 [191;271]	221 [192;278]	0.968
Hb[g/L]	126 [115;139]	128 [119;136]	0.419
ALT[U/L]	15.0 [12.0;21.0]	17.0 [14.0;23.0]	0.081
AST[U/L]	19.0 [15.0;23.0]	19.0 [16.0;23.0]	0.502
Scr[μmol/L]	70.0 [59.0;82.0]	71.0 [59.8;85.0]	0.798
UA[μmol/L]	324 [267;386]	322 [258;366]	0.309
eGFR(ml/min/1.73m2)	98.9 [84.9;108]	99.7 [84.7;112]	0.719
Cysc[mg/L]	0.97 [0.84;1.17]	0.99 [0.82;1.15]	0.828
ALB[g/L]	23.9 [20.5;28.4]	23.6 [20.4;28.6]	0.764
TCHO [mmol/L]	6.55 [5.46;7.90]	6.56 [5.78;7.87]	0.61
TG [mmol/L]	1.88 [1.15;2.90]	1.83 [1.42;2.49]	0.849
HDL [mmol/L]	1.32 [1.13;1.53]	1.35 [1.07;1.62]	0.619
LDL [mmol/L]	4.60 [3.34;5.99]	4.58 [3.57;5.97]	0.639
β2-MG[mg/L]	1.57 [1.22;2.24]	1.77 [1.12;2.32]	0.702
α1-MG[mg/L]	35.0 [29.0;43.0]	38.0 [29.0;46.0]	0.429
CRP[mg/L]	1.40 [0.40;2.50]	1.10 [0.50;2.27]	0.511
ESR[mm/h]	33.0 [18.0;64.0]	36.0 [17.0;57.0]	0.787
C3[g/L]	1.25 [1.10;1.45]	1.20 [1.08;1.44]	0.512
C4[g/L]	0.30 [0.26;0.35]	0.30 [0.26;0.35]	0.78
FT3[pmol/L]	3.94 [3.43;4.48]	3.83 [3.23;4.32]	0.122
FT4[pmol/L]	10.8 [9.72;11.8]	10.6 [9.40;11.8]	0.305
TSH[μIU/mL]	3.70 [2.56;5.66]	3.62 [2.38;5.89]	0.7
PLA2R[RU/mL]	40.3 [9.00;113]	59.3 [15.2;138]	0.264
24hTP[g]	5.00 [3.12;7.15]	4.24 [2.91;6.67]	0.18
CD4[/μl]	895 [647;1134]	867 [622;1124]	0.407
CD8[/μl]	440 [319;592]	440 [335;583]	0.723
CD4/CD8	1.94 [1.41;2.55]	1.96 [1.33;2.43]	0.384
Tubular atrophy:			0.711
0	50 (28.9%)	20 (26.7%)	
1	23 (13.3%)	14 (18.7%)	
2	50 (28.9%)	19 (25.3%)	
3	36 (20.8%)	18 (24.0%)	
4	14 (8.09%)	4 (5.33%)	
Interstitial fibrosis:			0.586
0	39 (22.5%)	23 (30.7%)	
1	8 (4.62%)	2 (2.67%)	
2	29 (16.8%)	11 (14.7%)	
3	97 (56.1%)	39 (52.0%)	

**Table 6:** Identification of potential risk factors in MMN by univariate and multivariate regression analysis.

Variable	Univariable		Multivariable	
	OR (95%CI)	p value	OR (95%CI)	p value
Age[years]	1.069 (1.04,1.098)	<0.001	1.055 (1.019,1.091)	0.002
BMI[kg/m2]	0.903 (0.831,0.981)	0.016		
SBP[mmHg]	1.011 (0.992,1.029)	0.26		
DBP[mmHg]	0.983 (0.955,1.012)	0.256		
History of hypertension	0.93 (0.504,1.716)	0.816		
Family history of malignancy	1.585 (0.672,3.737)	0.293		
WBC[109/L]	1.166 (1.025,1.326)	0.02		
PLT[109/L]	1 (0.996,1.005)	0.835		
Hb[g/L]	0.995 (0.976,1.016)	0.961		
ALT[U/L]	0.992 (0.961,1.024)	0.613		
AST[U/L]	1.004 (0.977,1.031)	0.774		
Scr[μmol/L]	1.015 (1.002,1.029)	0.024		
UA[μmol/L]	1 (0.997,1.003)	0.963		
eGFR (ml/min/1.73m2)	0.96 (0.944,0.977)	<0.001		
Cysc[mg/L]	12.952 (4.403,38.102)	<0.001	9.114 (2.386,34.815)	0.001
ALB[g/L]	1.051 (1.005,1.099)	0.029	1.072 (1.009,1.138)	0.024
TCHO [mmol/L]	0.915 (0.791,1.058)	0.229		
TG [mmol/L]	0.769 (0.596,0.993)	0.044		
HDL [mmol/L]	1.105 (0.552,2.211)	0.778		
LDL [mmol/L]	0.906 (0.78,1.052)	0.197		
β2-MG[mg/L]	1.933 (1.436,2.602)	<0.001		
α1-MG[mg/L]	1.012 (0.985,1.04)	0.392		
CRP[mg/L]	1.023 (0.994,1.052)	0.115		
ESR[mm/h]	1.007 (0.997,1.016)	0.184		
C3[g/L]	0.561 (0.205,1.535)	0.26		
C4[g/L]	2.722 (0.304,24.364)	0.371		
FT3[pmol/L]	1.287 (1.018,1.626)	0.035	1.722 (1.255,2.362)	0.001
FT4[pmol/L]	0.997 (0.979,1.014)	0.7		
TSH[μIU/mL]	1.024 (0.929,1.071)	0.306		
PLA2R[RU/mL]	1 (0.998,1.001)	0.741		
24hTP[g]	1.036 (0.946,1.135)	0.445		
CD4[/μl]	0.998 (0.997,0.999)	<0.001	0.998 (0.997,0.999)	<0.001
CD8[/μl]	0.998 (0.996,1)	0.014		
CD4/CD8	0.571 (0.392,0.832)	0.004		
Tubular atrophy	1.601 (1.259,2.037)	<0.001		
Interstitial fibrosis	1.359 (1.052,1.756)	0.019		

y = 2.336*Cysc+0.050*age-0.002*CD4+0.550*FT3+0.062*ALB-0.099*BMI-6.017.

### Model Discrimination and Calibration

**Figure 2:**
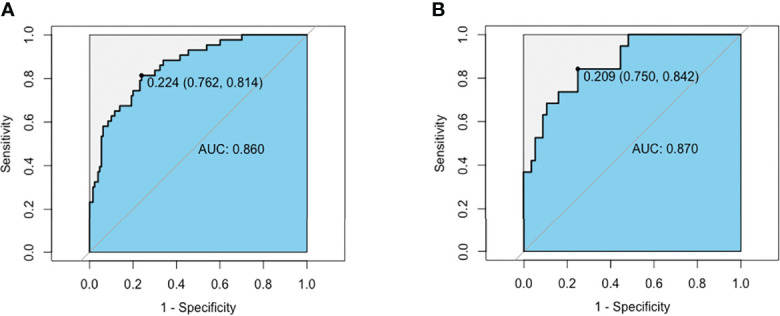
AUC of the MMN risk nomogram model. **(A)** ROC curve based on potential risk factors showing discrimination rate for MMN and IMN in training group. **(B)** ROC curve based on potential risk factors showing discrimination rate for MMN and IMN in test group.

**Figure 3:**
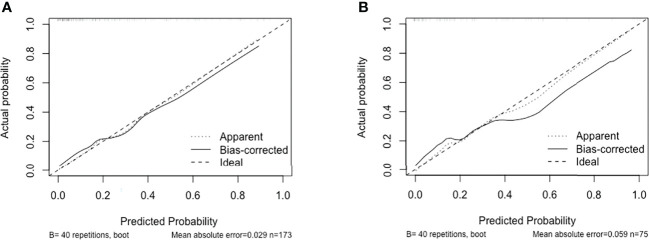
Calibration curve of discrimination nomogram in **(A)** training group or **(B)** test group. The x-axis represents the predicted probability of MMN. The y-axis represents the actual diagnosed MMN. The diagonal dotted line represents a perfect prediction by an ideal model. The solid line represents the performance of the nomogram, of which a closer fit to the diagonal dotted line represents a better prediction.

We performed receiver operating curve analysis to evaluate the combined diagnostic power of the regression-screened indicators. The area under the receiver operating curve, known as the C statistic, is considered to be a metric for assessing the validity of the model. We found that in training group, area under the curve (AUC) was 0.860 (cutoff value: 0.224, sensitivity: 0.762, specificity: 0.814; **Figure 2A**). We then verified validity of the model in test group. Results showed that AUC was 0.870 (cutoff value: 0.209, sensitivity: 0.750, specificity: 0.842; **Figure 2B**). These data demonstrated that the model had reliable predictive value. On drawing the calibration curve and evaluating the calibration ability of the model, its mean absolute error was found to be 0.029 (**Figure 3**); this indicated reliable calibration ability and small prediction errors of the model.

### Construction of the Nomogram

**Figure 4:**
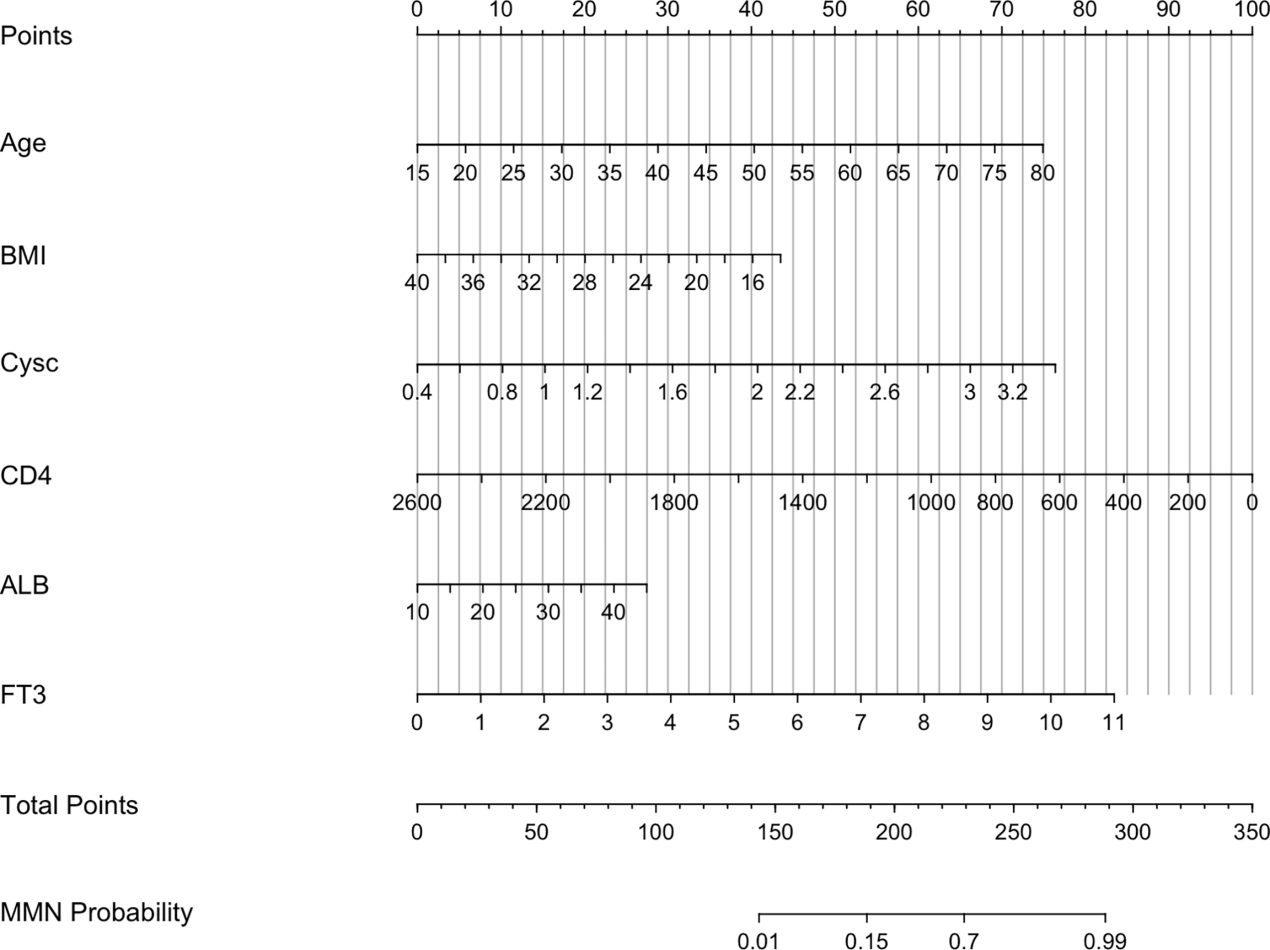
Nomogram predicting MMN. Cysc, Cystatin C; CD4, CD4+ T cells count; ALB, albumin; FT3, free triiodothyronine.

**Figure 5:**
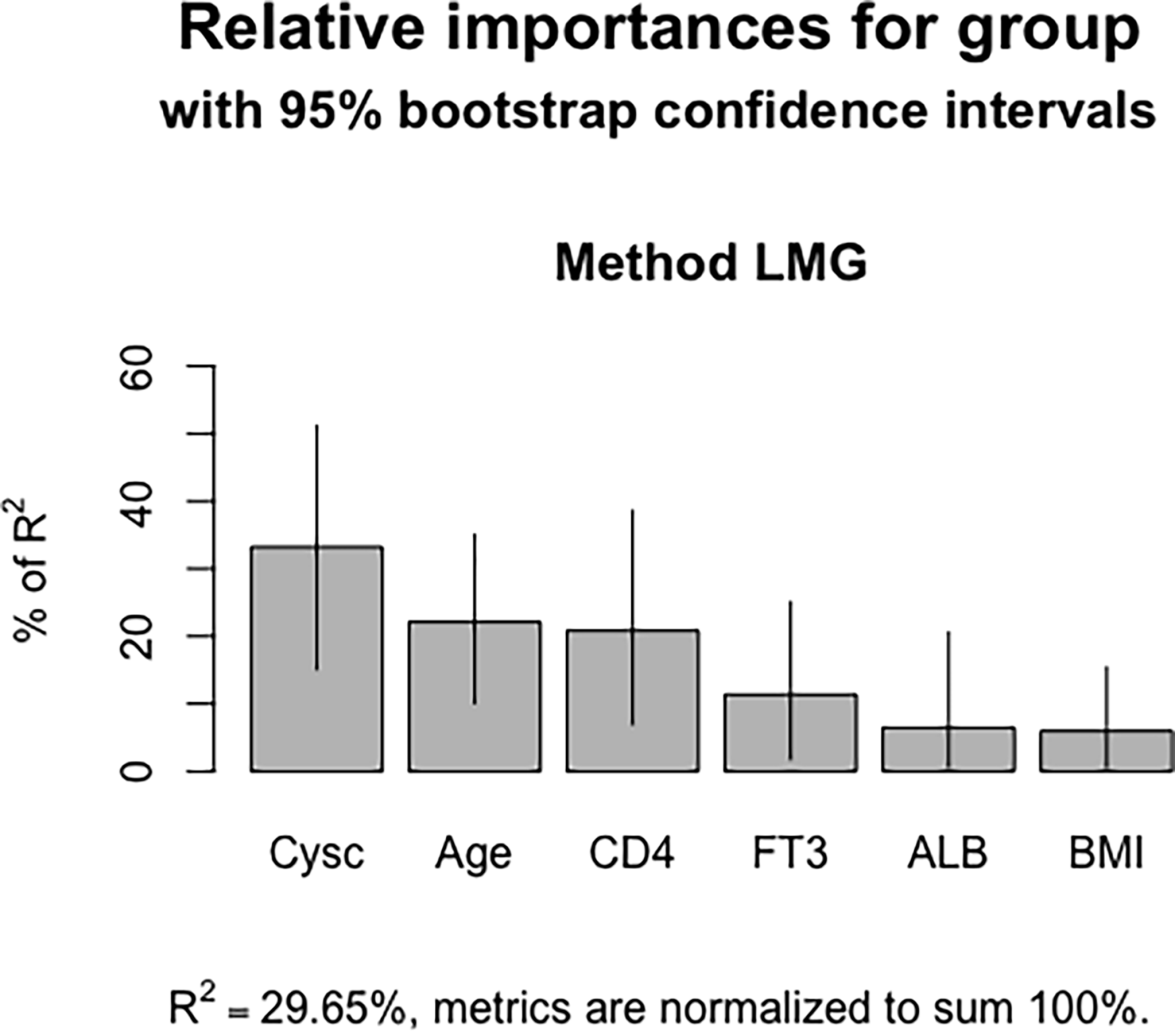
Coefficient of determination of the nomogram model. Cysc contributed the most in the model, followed by age, CD4+ T cell counts, FT3 and ALB.

A nomogram was constructed to determine the possibility of MMN more intuitively and to increase usability of the model. Using this nomogram, clinicians can calculate a patient’s total score by taking the score corresponding to each predictor and then reading the corresponding MMN likelihood based on the total score (**Figure 4**). In **Figure 5**, coefficient of determination (R^2^) plot showed that Cysc (33.2%) accounted for the highest proportion of variance in the model, followed by age (22.1%), CD4 (20.8%), FT3 (11.4%), and ALB (6.5%).

### Clinical Utility of the Model

**Figure 6:**
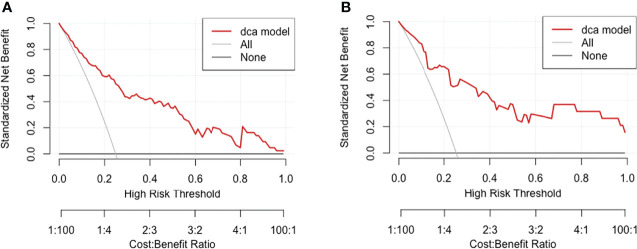
Decision curve analysis for MMN risk nomogram in **(A)** training group and **(B)** test group. The y-axis tested the net benefit. The thin gray line meant the assumption that all patients had MMN, while the thick red line represented the assumption that all patients had IMN. The dotted line represented the risk nomogram. In training group, the decision curve showed that if the threshold probability of a patient is between 0.01 and 0.93, using the nomogram in the present study to predict MMN adds more benefit.

The decision curve analysis is shown in **Figure 6**. Results showed that in threshold probability interval between 0 and 1, use of nomogram increased net benefit and had strong clinical utility in identifying MMN in training group.

## Discussion

We established a discriminant model for early screening and distinction between MMN and MN, to facilitate individualized precision treatment. We included 62 patients diagnosed as MMN, and 186 patients diagnosed as IMN. Model was constructed based on age, levels of Cysc, FT3, ALB, CD4+ T cell counts and BMI (AUC in training and test groups: 0.860 and 0.870, respectively). This model is of considerable significance for the early diagnosis of MMN, selection of treatment, and follow-up monitoring.

Distinction between MMN and IMN is critical owing to considerable differences in terms of treatment and prognosis. Some studies have discussed the early identification and screening of MMN. For instance, certain previous studies have indicated that PLA2R, THSD7A, and IgG subclasses may have the potential to aid recognition of MMN (10, 17). Although studies have focused on the pathological characteristics of MMN, the predominant type of deposited immune complex in MMN remains unclear. It is therefore essential to develop new methods for early screening of MMN. Our model is based on routine clinical indices, and has high diagnostic efficiency. High risk patients could be identified using this model, and it is expected to guide further monitoring or treatment.

As a quantitative tool for risk and benefit assessment, clinical prediction models can provide doctors, patients, and medical policy makers with more intuitive and rational information for decision-making (18, 19). Clinical prediction models have rapidly developed in renal disease, due to their scientific nature, accuracy, and simplicity. Examples of such models include those used for the distinction between MN and minimal change disease and between diabetic kidney disease and diabetes mellitus (20–22). However, MMN-related clinical models are currently lacking.

In this retrospective study, we collected 36 variables for regression analysis including demographic, clinical, and pathological indicators. Finally, we filtered out five simple variables for model construction. Based on their decreasing order of contribution, they were Cysc, age, CD4^+^ T cell count, FT3 levels, ALB levels and BMI. Cysc is a member of the cysteine protease inhibitor family and is used to evaluate glomerular filtration rate (23). We found that MMN cases had higher Cysc levels and lower eGFR, suggesting that these patients may have poorer renal function. Increasing experimental and clinical evidence indicates that Cysc is involved in the pathogenesis of various diseases including malignancy (24). But its specific role in the latter has not been clearly identified. Report indicates that it may promote or inhibit the growth and dissemination of tumor cells (25). The serum concentrations of Cysc can also be affected by tumor status. Owing to inflammation and cell turnover, Cysc synthesis is increased in immature dendritic cells in B-cell non-Hodgkin’s lymphoma; this makes it a less than ideal GFR surrogate (26, 27).

Our study found age of patients with MMN to be significantly higher. In agreement with our findings, Lefaucheur et al. found the mean age of patients with MMN to be significantly higher than that of those with IMN (28). It is worth noting that the association between age, malignancy, and MN may be exaggerated, as malignancy is itself strongly associated with advanced age. Therefore, it is unclear whether the relationship between age and MMN is causal or coincidental. However, in older patients with first-episode MN symptoms, there is a need to be highly vigilant regarding the possibility of any related malignancy. In older patients with cancer (especially those older than 65 years), it is particularly essential to note any changes in renal function, especially in those with solid tumors of lung and gastrointestinal tract (29).

CD4^+^ T cells perform a variety of functions in immune system. Abnormalities in their function are related to the occurrence and development of various diseases such as infection, tumor, and autoimmune conditions (30). Study has shown that number of CD4^+^ T cells in peripheral blood of patients with MN is significantly higher than that of healthy people (31). Interestingly, patients with malignant tumors have lower cellular immunity compared with healthy people. In this context, the exhaustion of CD4^+^ and CD8^+^ T cells is common in malignant tumors (32). The mechanism of CD4^+^ T cell exhaustion in malignant tumors and the rescue approaches constitute one of the current hotspots in field of tumor-related research. In our study, CD4^+^ T cell count was one of the meaningful indicators in prediction model. The baseline data showed that counts of CD4^+^ and CD8^+^ T cells and CD4^+^/CD8^+^ ratio were significantly lower in MMN than in IMN group; this was highly consistent with findings from previous studies on CD4^+^ T cells. Our regression model suggested that FT3 and ALB levels may also be associated risk factors for MMN.

In this study, there were significant differences in BMI between IMN and MMN groups. BMI was significant in the univariate logistic regression analysis, however the P value in the multivariate logistic regression was 0.062, greater than 0.05. Based on the important clinical significance of BMI for the diagnosis of various diseases, even if there is no statistically significant difference, we still believe that adding BMI, a routine indicator with important clinical value, into the model can improve the predictive ability of the model.

At baseline, there was a significant difference in anti-PLA2R antibody between IMN and MMN. However, after univariate/multivariate logistic regression analysis, PLA2R was not screened as an independent risk factor for MMN. Because of its very important significance in the diagnosis of IMN, we tried to incorporate it into the model based on clinical significance. The results showed that the diagnostic performance was partially improved, but contribution of PLA2R antibody in the model was small (**Figure S1**), which contributes 0.1% to the entire model. Therefore, the final model was constructed based on 6 conventional indicators of Cysc, FT3, and ALB, CD4+ T cell counts and BMI. Whether PLA2R can discriminate between IMN and MMN requires further validation in additional cohorts.

Previous studies have found differences in renal pathological characteristics of MMN and IMN, especially in terms of IgG deposition. It has been suggested that the high distribution intensity of IgG1 and IgG2 displayed by immunofluorescence is one of the predictors of MMN (5). More recent research suggests that deletions of IgG4 and anti-PLA2R are common in MMN, but other IgG subclasses do not differ significantly between two groups (4, 33). Histological features associated with renal pathology were also investigated in our study. We found no significant differences in terms of various pathological indicators including IgG and tubular atrophy/interstitial fibrosis. We speculate that the inconsistency in results may attributed to small sample size of previous studies. In this context, previous studies on MMN were mostly case reports or small studies with less than 20 cases. A recent study found that NELL1-associated MN is more frequently associated with malignancies than other known types of MN; it is therefore expected to be a diagnostic pathological marker for MMN (8). It is essential to explore and identify MMN-specific histochemical molecules or marker. This is also our direction for future research. This study had a retrospective design. Although it is currently difficult to obtain kidney tissue samples for immunohistochemistry, we expect the preliminary screening of patients with MMN using clinical data to provide further possibilities for investigation. We also reviewed the results of previous immunohistochemical experiments on kidney tissue in this study, including four indicators, namely, hepatitis B surface antigen, hepatitis B core antigen, amyloid P, and amyloid A. We found that the subjects included in study had tested negative for all four indicators. These were therefore not evaluated in the study. The findings suggest that the evidence for distinguishing MMN and MN based on pathology needs to be developed further.

Immunosuppression using alkylating agents in combination with corticosteroids or calcineurin inhibitor-based regimens has been the mainstay of treatment for IMN. However, the use of this treatment in patients with a tendency to develop tumors can aggravate disease. Expeditious screening for distinguishing MMN from IMN will therefore affect the choice of treatment and prognosis of patients. Our clinical prediction model, based on five laboratory indexes that are easy to collect and inexpensive to detect, is highly feasible. The model showed high diagnostic performance (AUC: 0.850) in distinguishing MMN and IMN. We suggest that patients with a high risk of MMN should be carefully monitored for the existence of tumors using appropriate tumor screening. Patients with high-risk score who have no tumor at the initial diagnosis should be carefully followed-up, since the sign of cancer may not be immediately obvious.

The model has certain limitations. The overall sample size was small, and external validation was not performed. Multi-center and multi-regional validation will be needed in future studies. Nevertheless, this is the first model to be constructed relating to this issue. We believe that it could help early screening and identification of MMN, thereby guiding treatment and follow-up.

## Data Availability Statement

The original contributions presented in the study are included in the article/**Supplementary Material**. Further inquiries can be directed to the corresponding authors.

## Ethics Statement

The studies involving human participants were reviewed and approved by the First Affiliated Hospital of Zhengzhou University Ethics Review Committee. The patients/participants provided their written informed consent to participate in this study.

## Author Contributions

ZZ and JS designed the study; TW and WY collected the data and drafted the paper; TW, FW and YZ analyzed the data and made the figures; ZZ and JS revised the paper. All authors approved the final version of the manuscript.

## Funding

This work was supported by the National Natural Science Foundation of China (Grant Nos. 81873611 and 8217033050), Science and Technology Innovation Team of Henan (Grant No. 17IRTSTHN020), 2020 key project of medical Science and Technology to JS.

## Conflict of Interest

The authors declare that the research was conducted in the absence of any commercial or financial relationships that could be construed as a potential conflict of interest.

## Publisher’s Note

All claims expressed in this article are solely those of the authors and do not necessarily represent those of their affiliated organizations, or those of the publisher, the editors and the reviewers. Any product that may be evaluated in this article, or claim that may be made by its manufacturer, is not guaranteed or endorsed by the publisher.
