# Supplementary figures and images for: Construction of a Nomogram Discriminating Malignancy-Associated Membranous Nephropathy From Idiopathic Membranous Nephropathy: A Retrospective Study
Source: Front Oncol. 2022 Jul 14;12:914092. doi: 10.3389/fonc.2022.914092 (PMC9329587; doi:10.3389/fonc.2022.914092)

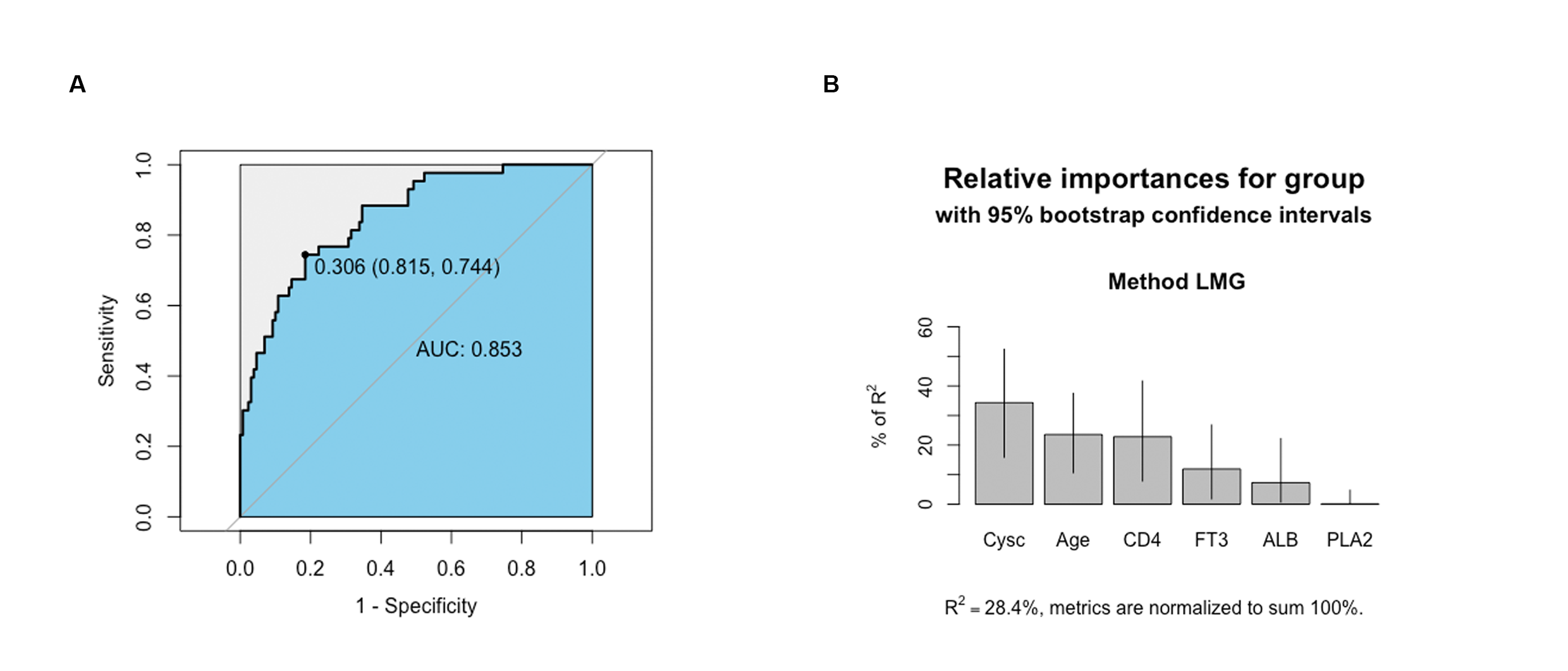

Supplement: Supplementary file 2 [file Image_1.tif]
